# How are people with orofacial clefts attended in northwest region of São Paulo state, Brazil?

**DOI:** 10.1590/1678-4685-GMB-2023-0167

**Published:** 2023-12-18

**Authors:** Marina Cristine Cano Francisquetti, Vera Lúcia Gil-da-Silva-Lopes, Agnes Cristina Fett-Conte

**Affiliations:** 1 Universidade Estadual Paulista, Instituto de Biociências, Letras e Ciências Exatas, Programa de Pós-Graduação em Biociências, São José do Rio Preto, SP, Brazil.; 2 Universidade Estadual de Campinas, Departamento de Genética Médica, Campinas, SP, Brazil.; 3 Faculdade de Medicina de São José do Rio Preto, Departamento de Biologia Molecular, São José do Rio Preto, SP, Brazil.

**Keywords:** Cleft lip, cleft palate, health care

## Abstract

Characterization of specific birth defects is essential for conducting scientific investigations, care and therapeutic strategies. This article describes demographic, clinical and genetic aspects, risk factors and access to treatment of Brazilian patients with orofacial clefts registered in a specialized collaborative center of the Brazilian Database on Craniofacial Anomalies (BDCA). We interviewed 70 individuals with typical orofacial clefts using a standard instrument from the database and subjected them to genetic testing. The patients were grouped as syndromic and non-syndromic. The majority of individuals were of lower middle class, native ancestry and syndromic. There was a significant difference in the type of clefts regarding gender. There was no significant difference between bilateral and unilateral, between the side affected, right and left, or familial recurrence related to type of oral cleft. The risk factor familial recurrence was significantly higher among non-syndromic cases. Etiological factors were identified or suggested in 62.5% of the syndromic cases. There was a delay in diagnosis and in access to treatment in most cases. We concluded that gender, native ancestry and low family income represent risk factors. Furthermore, the distribution by cleft types and gender is similar to previous studies. The results can guide scientific investigations and care policies.

## Introduction

Typical orofacial clefts (OFCs) are the most common craniofacial birth defects (BDs). They result from a complex interaction between multiple genetic and environmental risk factors. OFCs occur in isolation or in association with one or more BDs as part of syndromes (Leslie and Marazita, 2013). In general, syndromic cases of cleft palate (CP) are more common than syndromic cases of cleft lip (CL) or CL combined with CP (CLP) (Leslie and Marazita, 2013; Vyas et al., 2020).

Numerous variables have already been suggested as contributing to the genesis of OFCs, encompassing genetic interactions and environmental factors, such as intrauterine exposure to tobacco, alcohol, retinoic acid, and maternal diabetes (Leslie and Marazita, 2013). The genetic component is regarded as the predominant causal factor in 25 to 30% of the observed instances (Rodrigues et al., 2009). In addition, family recurrence is very common among patients with OFCs (Silva et al., 2018).

OFCs not only provoke aesthetic distortions of the face, but also may negatively affect the individual’s daily activities, especially feeding, hearing and speech, besides predisposing patients to infections and abnormal facial growth (Silva et al., 2018; Blum et al., 2023). The affected exhibit an elevated prevalence of mental health issues and elevated overall mortality rates across all life stages, even in developed nations, where proficient medical care is available (Beaty et al., 2016).

The incidence ranges depending on geographic location, and racial, ethnic, environmental, and socioeconomic characteristics (Salari et al., 2022). In Brazil, the estimated prevalence is 0.36 to 1.54 per 1,000 newborns (Neres et al., 2022). In this country, between the years 2020 and 2021, 3,288 cases of oral clefts were registered. The prevalence was 6.92 per 10,000 live births (Brasil, 2022). However, the prevalence varies between states and regions and few studies have been carried out to investigate these differences (Sousa and Roncalli, 2017). 

The diversity of clinical conditions related to OFCs, combined with the varying degrees of severity and potential associations with syndromes and other BDs results in specific therapeutic protocols that require the involvement of a multidisciplinary team of health professionals (Sousa and Roncalli, 2017). Patients with craniofacial anomalies need a long-term management and follow-up plan with professionals of various medical and surgical specialties. These individuals often need multiple surgical interventions and support therapies from childhood to early adulthood, such as speech therapy, occupational therapy and psychotherapy (Burg et al., 2016).

The genetic evaluation of individuals with OFCs is fundamental not only to improve the accuracy of diagnoses and to recognize comorbidities based on therapeutic planning and genetic counseling, but also to clarify the etiology and risk factors aimed at prevention (Burg et al., 2016; Gil-da-Silva-Lopes et al., 2019). Nonetheless, the epidemiological data required to assist public health care programs are scarce in Brazil, even in the state of São Paulo, where there are many referral health centers and research centers. Despite providing assistance, these institutions by itself do not present a health policy capable of modifying the current scenario (Ise et al., 2019; Gil-da-Silva-Lopes *et al.*, 2020). 

Based on the needs identified in the Brazilian population, a project called Brazil’s Craniofacial Project (BCFP, 2022) was created in 2003 to bring together collaborative centers from different regions of the country - research centers, universities and hospitals (Gil-da-Silva-Lopes et al., 2020). In addition to the diagnostic investigation, partnered with the World Health Organization (WHO), the project developed and validated the Brazilian Database on Orofacial Clefts with clinical and family data (Monlleó et al., 2013). This project was expanded, giving rise to the Brazilian Database of Craniofacial Anomalies (BDCA), which uses a broad standardized protocol following WHO (2011) recommendations for data collection and the assessment of patients with craniofacial anomalies. A Web-based application called CranFlow® (registered by the National Institute of Industrial Property, number BR 51 2015 000550 2) was developed to collect and manage etiological, epidemiological and the phenotypic spectrum data of different orofacial anomalies of populations from various regions. This tool created conditions for more effective multiprofessional participation, today it has 12 collaborating centers involving different health professionals in the Northeastern, Southeastern and Southern regions of Brazil (Volpe-Aquino et al., 2018). 

One of these collaborative centers is the Hospital de Base (HB) of São José do Rio Preto (SJRP), a university hospital where most patients receive health care from the Brazilian Unified Health Care System (SUS). It is located in São José do Rio Preto, a Brazilian municipality in the northwest region of São Paulo state, seat of the Metropolitan Region of São José do Rio Preto, which has 37 municipalities.

This study presents data on the demographic, genetic and clinical profiles, risk factors and access to treatment of syndromic and non-syndromic individuals with OFCs attended at a new specialized collaborative center of the BDCA/CranFlow® located in a city of São Paulo State, Brazil, named BDCA/CranFlow®/HB/SJRP.

## Methods

The Research Ethics Committee of the Faculty of Medicine of São José do Rio Preto approved this retrospective descriptive cohort study (CAAE: 76875117.7.0000.5415). We recruited patients at Hospital de Base (HB-FAMERP/FUNFARME), a tertiary care hospital located in São José do Rio Preto, São Paulo. All adult subjects and legal guardians of underage patients signed a Written Informed Consent Form.

Individuals with OFCs who previously attended the Genetic and Otorhinolaryngology Outpatient Clinic of HB were invited to be registred in the database. About 100 patients were invited, of which 30 did not accept the invitation for different reasons, or scheduled but did not attend the interview. Seventy cases with OFCs aged from one month to 47 years were enrolled and interviewed to collect data. They were registered in the BDCA/CranFlow®/HB/SJRP (https://www.fcm.unicamp.br/fcm/cranio-face-brasil/projeto-cranio-face-brasil). 

The adopted inclusion criteria comprise individuals of any age with CLs, CPs or CLPs in isolation or as a part of a syndrome, or with multiple BDs and random additive defects (Monlleó et al., 2013). The exclusion criteria comprised individuals with atypical OFCs (median, oblique, transverse, and others), holoprosencephaly and uvula cleft, according to the criteria recommended for exclusion of patients of the current database at the time of the study. 

We conducted interviews with the affected individuals and/or their parents, following the guidelines of the BDCA, which includes 192 items. These items encompass information related to demographics, origin, gender, ethnic background, gestational and neonatal periods, delivery, family recurrence, exposure to teratogenic agents, parental age, maternal diseases, clinical characteristics, and the results of complementary and genetic exams. Data were collected through interviews and patients’ clinical data, medical histories, and exam results, according to the needs of each patient. We also analyzed the time of diagnosis, the age at which treatment commenced (irrespective of type, if related to the cleft), access to the initial surgical intervention, and support therapies.

At the HB Center, the surgical protocol includes the following steps: lip correction (cheiloplasty) is typically performed between 3 to 6 months of age or when the patient’s weight exceeds 5 kilograms. Palatal correction (palatoplasty) is carried out between the ages of 12 to 18 months. Bilateral cleft individuals undergo bilateral lip correction at 4 months of age. Subsequent to the surgical interventions, orthodontic and maxillary orthopedic monitoring continues until post-puberty, in addition to rhinoplasty.

Some demographic questions, including family income, were not initially included in the database items. For this reason, we could not obtain from all patients, what was a limitation of our study. We transformed the information of patients about family income to minimum wage. In addition, some affected individuals and/or their parents, especially older cohort members, did not remember some of the requested information, which is why in the Results section there are variable sample numbers according to the factor investigated.

The data were coded based on the International Clearinghouse for Birth Defects Surveillance and Research (ICBDSR, 2021). The Human Phenotype Ontology system (HPO, 2022) was used to describe clinical signs identified in patients. Moreover, the International Classification of Diseases (ICD-10) and Online Mendelian Inheritance in Man (OMIM) were used to code the diagnoses. CranFlow® meets the established safety regulations for medical data (Volpe-Aquino et al., 2018).

Group definitions also followed the criteria employed in the BDCA/CranFlow®. The subdivision into syndromic and non-syndromic OFCs depended on the presence of associated structural and developmental congenital defects (CDs). OFCs were considered syndromic when there was any combination of two or more major defects due to a single previously demonstrated or highly probable etiological factor based on its recurrence in a number of cases; when not associated with other BDs, OFCs were considered non-syndromic or isolated OFCs (Monlleó et al., 2013). 

For laboratorial analysis, we collected 15 mL of peripheral blood in tubes containing heparin or EDTA from the patients. Depending on the indication, the sample with heparin was used for GTG-band karyotyping (in 57 patients) and Fluorescence *in situ* Hybridization (FISH) (in 21 patients), both of which were performed following the standard routine of the Genetics Laboratory (HB/FUNFARME). EDTA treated samples were used in molecular studies and sent to the DNA Biorrepository of Individuals with OFCs in the coordinating center (UNICAMP/Campinas). Multiplex Ligation Probe-dependent Amplification (MLPA) (17 patients) and Array Comparative Genomic Hybridization (a-CGH) (13 patients) were performed following the manufacturer's instructions and based on diagnostic hypotheses. Many patients have had more than one genetic test.

The results are presented as absolute numbers (n) and frequencies (%). Exploratory data analyses using summary measures (mean, standard deviation, minimum, median, maximum, frequency and percentage) were performed and differences compared employing Chi squared analysis or Fisher’s exact test. All measures were calculated with a 95% confidence interval and significance was established as p-values < 0.05. All statistical analyses were performed using the SAS System for Windows (Statistical Analysis System, version 9.4, SAS Institute Inc.). 

## Results

**Figure 1 -:**
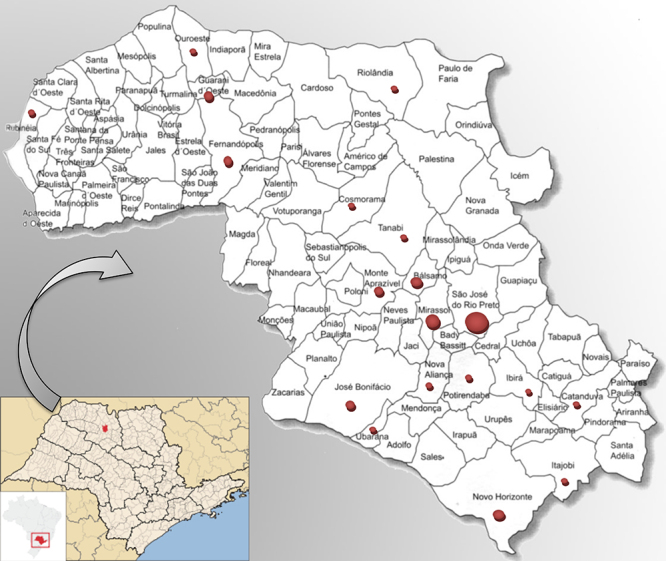
Map of the state of São Paulo highlighting the region of São José do Rio Preto and the main towns where the 70 individuals under study live. The size of the circle is proportional to the number of cases.

Of 70 subjects, 40% lived in São José do Rio Preto, while 60% lived in other municipalities of the region (Figure 1).

Thirty-eight participants informed their family income (Table 1) as minimum wages (MW). The average family income was 2.07 MW per month - about US$517 (mode of 1 MW; median of 2 MW), corresponding to lower middle class in 73.7% of families.

**Table 1 -:** Distribution of individuals stratified by family income.

MW	Number of families	Frequency (%)
1	14	36.84
1.5	2	5.26
2	12	31.58
2.5	1	2.63
3	3	7.90
4	4	10.53
5	2	5.26

^a^ MW = minimum wage.

Information on ethnic ancestry was obtained from 53 mothers and 48 fathers. A total of 40 (75.5%) mothers and 33 (68.7%) fathers self-declared themselves as native ancestry (Latin European + African + Indigenous). Therefore, most patients were of native descent.

The type of OFCs were classified in 57 cases. Some patients were attended at the center only as adults, after surgical interventions, and did not present previous medical data, which made it difficult to identify their type of cleft. Table 2 shows the type stratified by gender, laterality, presence or absence of syndrome and family recurrence. There was a significant difference (p-value: 0.0379) in the type of clefts regarding gender. Males were more affected (32; 56.1%) and presented more CLPs while women presented more CPs. There was no significant difference between bilateral and unilateral cases or between the side affected, right and left. Most of the affected individuals were syndromic (p-value: 0.0201) and there was no significant difference related to familial recurrence.

**Table 2 -:** Distribution of individuals according to the type of oral cleft stratified by gender, laterality, classification and familial recurrence.

Variable	Category	CL n (%)	CP n (%)	CLP n (%)	Total n (%)	p-value
Gender (n:57)	Female	4 (50.00)	13 (65.00)	8 (27.60)	25 (43.90)	0.0379^1^
	Male	4 (50.00)	7 (35.00)	21 (72.40)	32 (56.10)	
Laterality (n:37)	Unilateral - Right	3 (37.50)	-	6 (20.70)	9 (24.40)	0.3483^1^
	Unilateral - Left	3 (37.50)	-	8 (27.60)	11 (29.70)	
	Bilateral	2 (25.00)	-	15 (51.70)	17 (45.90)	
Classification (n:57)	Non-syndromic	2 (10.00)	3 (15.00)	15 (75.00)	20 (35.10)	0.0201^1^
	Syndromic	6 (16.20)	17 (45.90)	14 (37.80)	37 (64.90)	
Familial recurrence (n:41)	Yes	3 (37.50)	3 (30.00)	11 (47.80)	17 (41.50)	0.6925^1^
	No	5 (62.50)	7 (70.00)	12 (52.20)	24 (58.50)	

^a^ Cleft Lip (CL).

^b^ Cleft Palate (CP).

^c^ Cleft lip with cleft palate (CLP).

^d^ n = number of individuals.

^1^ *p*-value = Fisher’s exact test.

From 70 subjects, 48 (68.6%) were syndromic and 22 (31.4%) were non-syndromic. The diagnosis/etiologic factor were identified in 30 (62.5%) of the syndromic cases and the other 18 (37.5%) were classified as multiple birth defects/unknown. Genetic causes (monogenic or chromosomal etiology) were more frequent (23 cases), with predominance of 22q11.2 deletion syndrome (30.4%) (Table 3). A 5:2 ratio was observed of affected women and men with 22q11.2 deletion. 

**Table 3 -:** Diagnosis/etiology and diagnostic evidence of syndromic cases.

Diagnosis/Etiology	N	Diagnostic evidence
MBDs/unknown	18	Clinical
Chromosome 22q11.2 deletion syndrome/genetic	7	FISH and MLPA
Fetal alcohol syndrome/environmental	3	Clinical
Complex chromosomal rearrangement/genetic	2	Karyotype
Amniotic band syndrome/environmental	1	Clinical
Adams-Oliver Syndrome/genetic	1	Clinical
Branchio-Oculo-Facial syndrome (BOFS)/genetic	1	Clinical
Chromosome 3p26.1 deletion/genetic	1	a-CGH
Chromosome 16p11.2 deletion syndrome/genetic	1	a-CGH
Craniofrontonasal syndrome/genetic	1	Clinical
Disruption by maternal diabetes/environmental	1	Clinical
Dubowitz syndrome/genetic	1	Clinical
EEC syndrome/genetic	1	Clinical
Fanconi syndrome/genetic	1	Karyotype/DEB
Ischiocoxopodopatellar syndrome/genetic	1	Clinical
Moebius syndrome/multifactorial	1	Clinical
Noonan syndrome/genetic	1	Clinical and Molecular
Pierre-Robin syndrome/multifactorial	1	Clinical
Optiz G/BBB syndrome/genetic	1	Clinical
Orofaciodigital Syndrome type 1/genetic	1	Clinical
Van der Woude syndrome/genetic	1	Clinical and Molecular
Wolf-Hirschorn syndrome (4p-)/genetic	1	Karyotype and FISH

^a^ N = number of syndromic cases.

^b^ MBDs = multiple birth defects.

^c^ FISH = Fluorescence In Situ Hybridization.

^d^ MLPA = Multiplex Ligation Probe-dependent Amplification.

^e^ a-CGH = Array-Comparative Genomic Hybridization.

^f^ DEB = diepoxybutane.

Possible risk factors for OFCs were observed in both groups (Table 4). Male and female patients were equally affected among the syndromic patients. Advanced age and parental consanguinity were observed in the same number of cases of both the syndromic and non-syndromic groups. In addition, there was no significant difference between groups regarding exposure to teratogenic agents, total number of pregnancies, gestational age and maternal disease. Familial recurrence was the only risk fator that significantly differed between the group; it was significantly more frequent in non-syndromic subjects (p-value: 0.0362).

**Table 4 -:** Frequency of risk factors in syndromic and non-syndromic groups.

Risk Factor	Category	Syndromic n (%)	Non-syndromic n (%)	p-value
Gender (n:70)	Female	24 (50.00)	8 (36.40)	0.2877^2^
	Male	24 (50.00)	14 (63.60)	
Paternal age at birth (n:51)	≥ 40	4 (12.10)	4 (22.20)	0.4297^1^
Maternal age at birth (n:56)	≥ 35	3 (8.10)	3 (15.80)	0.3971^1^
Consanguinity (n:62)		1 (2.40)	1 (4.80)	-
Alcohol (n:59)		8 (21.60)	1 (4.50)	0.1337^1^
Tobacco (n:59)		6 (16.20)	3 (13.60)	1.0000^1^
Illicit drugs (n:59)		2 (5.40)	0 (0.00)	-
Exposure to other teratogens^*^ (n:54)		14 (41.20)	7 (35)	0.6530^2^
Total number of pregnancies (n:69)	1	15 (31.30)	5 (23.80)	
	2 to 4	30 (62.50)	13 (61.90)	0.5176^1^
	≥ 5	3 (6.30)	3 (14.30)	
Gestational age (n:43)	Term	16 (66.70)	14 (73.70)	0.6188^2^
	Preterm	8 (33.30)	5 (26.30)	
Maternal disease (n:59)	Diabetes	2 (5.30)	3 (14.30)	0.3365^1^
	Epilepsy	1 (2.60)	1 (4.80)	-
	Obesity	0	2 (9.50)	-
	Others	7 (18.40)	2 (9.50)	0.4687^1^
Familial recurrence (n:45)		7 (26.90)	11 (57.90)	0.0362^2^

*Others teratogens: solvents, medicines, domestic and/or occupational exposure to insecticides, herbicides and pesticides.

^1^ *p*-value = Fisher’s exact test.

^2^ *p*-value = Chi-square.

There was no significant difference between the two groups regarding the stage at diagnosis (prenatal vs. postnatal), age at beginning of specialized treatment, first surgical correction of OFCs and access to speech therapy (Table 5). All the cases of CP of both groups received diagnosis in the postnatal period. Regarding CLPs, most syndromic cases (70%) were diagnosed during the prenatal period, while most non-syndromic cases of CL and CP (66.67%) were diagnosed in the postnatal period. Most syndromic and non-syndromic patients underwent specialized treatment within the first month of life; 24.4% of cases started treatment at 24 months or more of life. Of the 57 cases in which clefts were characterized, 12 (21.05%) were not submitted to surgical intervention before the age of two years, nine of which were syndromic and three non-syndromic. 

**Table 5 -:** Stage at diagnosis, treatment timing and therapy received in syndromic and non-syndromic groups.

Variable	Category	Specification	Syndromic n (%)	Non-syndromic n (%)	p-value
Stage at diagnosis (n: 37)	CL	Prenatal	2 (40.00)	-	-
		Postnatal	3 (60.00)	2 (100)	
	CP	Prenatal	-	-	-
		Postnatal	6 (100)	2 (100)	
	CLP	Prenatal	7 (70.00)	4 (33.33)	0.0868^2^
		Postnatal	3 (30.00)	8 (66.67)	
Age at beginning of treatment (n: 41)	No		1 (3.30)	0	-
	≤ 1 month		15 (50.00)	5 (45.50)	
	≤ 12 months		6 (20.00)	0	
	13-23 months		0	1 (9.10)	
	≥ 24 months		5 (16.70)	5 (45.50)	
First surgical intervention ≤ 24 months	CL (n: 8)	Yes	3 (60.00)	2 (100)	-
		No	2 (40.00)	-	
	CP (n: 20)	Yes	2 (33.33)	2 (100)	-
		No	4 (66.67)	-	
		Lip correction	5 (50.00)	4 (33.33)	0.5681^1^
	CLP (n: 29)	Palate correction	-	-	
		Both	2 (20.00)	5 (41.67)	
		None	3 (30.00)	3 (25.00)	
Support therapy	Speech therapy (n:41)	Yes	19 (76.00)	9 (56.30)	0.1849^2^
		No	6 (24.00)	7 (43.80)	
	Others* (n:41)	Yes	14 (56.00)	2 (12.50)	0.0053^2^
		No	11(44.00)	14 (87.50)	

^*^ Physiotherapy, Occupational Therapy, Equine therapy and Hydrotherapy.

^a^ n = number of individuals.

^1^ *p*-value = Fisher’s exact test.

^2^ *p*-value = Chi-square.

The average age at the first surgical intervention in CL syndromic cases was 10.3 months and 11 months in non-syndromic cases. The average ages for surgical correction of CP were 18 months and 39 months for syndromic and non-syndromic cases, respectively. On considering individuals with CLP in both groups, the average age was 21.8 months for CL correction and 110.4 months for CP correction. Within this series, two adolescents have not undergone CLP corrective surgeries yet. The reasons that prevented surgical intervention included: low weight at the time indicated for the surgical procedure, lack of referral to specialized centers, long waiting list in the public referral hospital, and change of address without it being updated in the hospital, thereby preventing contact (data not shown in tables).

Most patients of both groups had access to support therapy, especially speech therapy, which was indicated in all cases. However, 31.7% of patients in general had no access to this treatment. There was a significant difference between syndromic and non-syndromic patients regarding access to physiotherapy, occupational therapy, equine therapy and hydrotherapy, which was greater among syndromics (p-value: 0.0053).

## Discussion

About 185,000 children are born with birth defects in the US and about 8 million worldwide each year (Reece, 2012). However, studies in underdeveloped or developing countries are scarce, generally hospital-based, without direct evaluation of the child and performed by analyzing medical records or mandatory official records, with underreporting of cases. Studies of specific congenital defects, such as OFCs, are even scarcer with prevalences that vary by region and federative units (Sousa and Roncalli, 2017). For example, in Acre State (Northern Brazil) the prevalence is 6.68: 10,000 births, while in Rio Grande do Sul State (Southern Brazil) it is 9.59: 10,000 (Brasil, 2022; SINASC, 2022). According to SINASC (Sistema de Informação sobre Nascidos Vivos), between 2020 and 2021, a prevalence was observed of 7.94 and 5.16 per 10,000 live births in the states of São Paulo and Rio de Janeiro, respectively (Brasil, 2022; SINASC, 2022). However, it is estimated that the lack of notification in the national birth registry is 49.9% (Souza and Raskin, 2013).

According to the last census of the Brazilian Institute of Geography and Statistics, São José do Rio Preto has 408,258 inhabitants (IBGE, 2022), with 5,097 births occuring in 2018 (DRSXV, 2019). Data of 70 individuals added to the database over five years are presented herein, which means that a representative number of OFC cases were studied. Although the sample comprises individuals from one of most populous regions of Brazil, it should be kept in mind that this was a voluntary research. Thus, the proportion of phenotypes of cleft as well as data of consanguinity or recurrence do not represent the population prevalences.

Most subjects were from São José do Rio Preto and surroundings towns, as the city has a center of excellence for the treatment of patients who need complex and highly specialized procedures covered by the Brazilian national health care system. Furthermore, Hospital de Base has the only Genetics Service in the region, and so it attracts individuals with OFCs from the Southeastern Region of the country (Hospital de Base de São José do Rio Preto, 2018). 

The results on family income, measured in minimum wages, revealed the predominance of less favored socioeconomic classes. This data was not obtained for all the individuals in the sample, what is a limitation, because it was only considered in the later stages of the implementation of the BDCA. 

There are controversies in the literature about the correlation between socioeconomic factors and orofacial clefts due to the methodological diversity used in different study models (Deguen et al., 2016). Some studies have shown that most OFC cases occur in children living in low socioeconomic conditions (Rodrigues et al., 2009). However, this alleged correlation does not necessarily imply that the socioeconomic status is an independent risk factor for craniofacial anomalies, but it certainly favors greater exposure to other risk factors, such as poor nutrition, and a greater difficulty to access healthcare services increasing the risk of maternal-fetal diseases. In fact, it is also possible to explain the predominance of economically disadvantaged families, considering that patients were treated in a university hospital under the Brazilian Unified Health Care System (SUS). 

Ethnicity is a risk factor for orofacial clefts. Palate clefts, for example, are more frequent in Asians (Burg et al., 2016). The data obtained during the interview on the ancestry of the parents of patients showed that most were of native descent, which, according to the criteria used by the BDCA, is a miscegenation of Latin American, African and Indigenous origins. One can justify this finding by the intense miscegenation of different ethnic groups in the country. Even after centuries, traces of colonization remain intact in the genome of the Brazilian population. Levels of miscegenation may vary by region with the most common mixing being between Afro-Brazilians and Caucasians (Rego Borges *et al.*, 2015).

This is not a population-based study and care must be taken when comparing our results. However, the distribution of individuals by type of oral clefts, gender, laterality, classification as syndromic and non-syndromic, and familial recurrence were greatly similar to those previously reported rates. 

We observed that males were more affected and presented more CLPs while women presented more CPs. Gender-dependent susceptibility of clefts lacks elucidation. Blanco et al. (2001) proposed that male susceptibility to CLs and CLPs may be associated to a certain extent with a *MSX1* gene variation mapped on chromosome 4. On the other hand, the association of CPs with female gender may be associated with embryo closure time of the secondary palate, which occurs later in women than in men (Souza and Raskin, 2013). However, recent genome-wide interaction studies identified gender-specific risk alleles for non-syndromic orofacial clefts (Carlson et al., 2018). 

As CL and CLP may be pathogenetically distinct, researchers should analyze them separately (Harville et al., 2005; Souza and Raskin, 2013). We found a predominance of CLPs, a small predominance of unilateral clefts over bilateral clefts, greater impairment of the left side when compared to the right, a statistical association between CLPs and male gender and between CPs and female gender. In addition, individuals with CLPs exhibited bilateral dysfunction more frequently than those with CLs. These findings are similar to those previously described (Monlleó et al., 2015; Alfwaress et al., 2017). 

About 70% of the cases of orofacial clefts are non-syndromic, while the remaining 30% from Mendelian inheritance, chromosomal alterations and teratogenic conditions. In addition, most cases of OFCs are isolated, while in syndromic cases it is possible to observe a greater association with CPs, as in this study (Monlleó et al., 2015; Woo, 2017; Vyas et al., 2020). However, our data showed that 68.6% of cases were syndromic and 31.4% were non-syndromic. This is not a population study with the objective of investigating the prevalence, and the frequency of syndromic and non-syndromic cases was calculated in a sample with participants who were initially attended at a genetic service, where syndromic cases are more frequently seen. In addition, many invited families did not agree to participate in the study, so that inclusion by type was random and beyond the control of the researchers.

Currently, over 400 genetic disorders are reported to include CL or CLP (OMIM, 2021; Lace et al., 2022). They are observed in syndromes with unknow prevalence as Craniofrontonasal and Escobar, rares as Van der Woude (1: 35,000-100,000) and Orofaciodigital (1: 50,000-250,000), and in that with higher prevalent as 22q11.2 deletion syndrome (1: 4,000), Pierre Robin (1: 8,500-14,000) and Charge (1: 8,500-10,000). But the most common forms of syndromic oral clefts are Van der Woude syndrome and 22q11.2 deletion syndrome (Burg et al., 2016). In this study many patients with syndromic OFCs showed some of these conditions, as observed in previously reports (Jaruratanasirikul et al., 2016).

Although several genetic alterations translate as peculiar phenotypes with signs and symptoms that enable clinical diagnosis, other cases are harder to diagnose. There is a need to identify cleft-causing mutations in all patients of a sample, which would require several high-cost molecular tests; we did not perform these tests in this study due to financial limitations. Diagnoses based on clinical examinations reinforce the importance of genetic evaluations by a specialized professional and the genetic follow-up of patients with OFCs. Nonetheless, this is not always enough. In this study, 18 cases with normal karyotypes had no diagnosis of specific affections despite the presence of multiple BDs, reinforcing the importance of carrying out more specific genetic tests when studying OFCs without etiological diagnosis. However, it is worth highlighting that in countries with poor investment in health, such as Brazil, the population’s access to high-cost exams is highly limited, even in referral centers (Monlleó et al., 2015).

The obtainment of karyotype, FISH and a-CGH, considering the genetic criteria of indication in each case may result in etiological clarification in many cases, as we observed. For example, genetic causes were more frequent in this study, with predominance of the Chromosome 22q11.2 deletion syndrome (SD22q11.2), as expected as it is the most common microdeletion syndrome; it is most frequently associated with CPs. Resulting from a heterozygous deletion of chromosome 22q11.2, it is the CNV (copy number variation) most commonly associated with OFCs and affects men and women equally (Repetto et al., 2014; Vangkilde et al., 2016; Lin et al., 2017). The 5:2 ratio of affected women to men have been casual in this study due to the small sample size.

Alcohol consumption was the most frequently observed isolated environmental risk factor for OFCs. The effect of alcohol consumption during pregnancy can result in Fetal Alcohol Syndrome (FAS). This syndrome interferes negatively in the development of the fetus and may lead to several BDs including OFCs (Yin et al., 2019). In developing countries, such as Brazil, there are still few studies on the teratogenic risks of alcohol consumption in the gestational period (Silva et al., 2018). 

Other risk factors include advanced paternal age (≥ 40 years) and advanced maternal age (≥ 35 years), maternal smoking, poor nutrition, metabolic status (diabetes and obesity) and exposure to other teratogens, such as illicit drugs, solvents, medicines, domestic and occupational exposure to insecticides, herbicides and pesticides during the gestational period (Martelli et al., 2015), many of which were observed in the current sample, although in few cases. 

Advanced maternal age is a risk factor for BDs, including OFCs, due to anomalies resulting from numerical chromosomal changes. Advanced paternal age also increases the risk of children with OFCs, especially CPs (Herkrath et al., 2012; Burg et al., 2016). Some studies also associate maternal diseases with the etiology of OFCs, especially with gestational diabetes, which is an etiological factor in several congenital anomalies, including CLP (Martinelli et al., 2020). 

Advanced age and parental consanguinity were observed at similar frequencies in syndromic and non-syndromic cases in this study with no significant difference related to the total number of pregnancies. Familial recurrence was the only risk factor that differed significantly between the groups, more significantly in non-syndromic subjects. This was expected, considering that syndromic cases are usually sporadic in genealogies, while there is strong family aggregation ascribed to the multifactorial threshold model of inheritance that is characteristic of non-syndromic OFCs (Silva et al., 2018).

Parental consanguinity and familial recurrence are risk factors that called the attention of professionals because of the genetic component in the etiology of OFCs and the risk of recurrence in first- and third-degree relatives when compared to the background population (Gil-da-Silva-Lopes and Monlleó, 2014). Although most CL, CP and CLP cases are sporadic within a family, a positive familial history occurs in about 26% of cases. The chance of the child with a sibling with CL/P also being affected is about 30-40 times higher than the risk in the general population (approximately 0.1%). In addition, children born from consanguineous couples present a higher risk of occurrence (Burg et al., 2016; Silva *et al.*, 2018). 

According to a city-wide population-based observational study performed in São José do Rio Preto, of 5,204 children born between March 2011 and February 2012, gestational diabetes was observed in ten cases, alcohol consumption during pregnancy in 28, consanguinity in three, advanced maternal age (35 years or more) in 451 and advanced paternal age (40 years or more) in 327 cases (Oliveira-Brancati et al., 2020). 

The time of intervention is essential in order to reach the best outcomes. But surgery of cleft palate is a difficult challenge for all surgeons because patients are very different regarding complexity, especially syndromics. There is need for specialized, multidisciplinary, long-lasting treatment with surgical interventions at appropriate times. The WHO (2011) recommends care by a multidisciplinary and highly specialized team and including a genetic evaluation. However, few specialized centers offer this service, approximately 28 throughout Brazil (CNES, 2022), a low number considering the country’s population. In addition, not every center has sufficient material and human resources, and not all patients have access to these centers, which indicates the need to change health policies (Monlleó et al., 2015). 

In fact, many of the patients in this study were born without diagnoses, which raises doubts, causes anxiety and scares of parents. Amstalden-Mendes et al. (2011) believe it is fundamental to provide perinatal and postnatal counseling to parents to clarify all these issues and to promote access to relevant information and treatment.

There are different surgical protocols (Gatti et al., 2017), however, lip correction and closure surgery should be carried out from 3 to 6 months of age and palate correction should occur from 9 to 12 months if possible and not exceeding 18 months of age (Silva et al., 2018; dos Santos and de Oliveira, 2021). At these ages, anesthesia is safer, the repair is more accurate, and parents are more likely to accept malformations. The ideal correction must ensure facial growth and speech development (Silva *et al.*, 2018).

In this study, the average age of the first surgical intervention in all types of OFCs, was higher than that recommended by different surgical protocols, and is also higher than the protocol used by the HB center. This delay can indicate the precarious access to surgical treatment and inadequate planning of treatment even in referral hospitals. Our data corroborate those recently reported by Ise et al. (2019) which associate diagnosis and treatment delays in patients receiving late primary repairs. In contrast, they may also represent not only a lack of access to services, but also the patients’ lack of awareness of the importance of early treatment and the lack of public policies focused on the identification of cases and referral to specialized services. According to a multicontinent study, the patient travel costs, lack of patient awareness, and lack of financial support are the most commonly reported barriers to providing CLP care. Policy makers should consider identified barriers to improve healthcare equity and increase the quality of CLP care delivered around the world (Massenburg et al., 2016).

The delay of treatment increases morbidity and impairment of the individual’s development, including difficulties to eat, speak, and listen, psychological problems, and even social exclusion (Silva et al., 2018). These impairments were identified in the patients of this study. Many exhibited language delay, motor delay, and behavioral delay affecting the development.

In some cases, insufficient sucking and the ingestion of insufficient amounts of food do not guarantee the individual’s nutritional needs and do not promote a caloric surplus for weight gain, thus not allowing the patient to perform surgical correction at the appropriate time (Amstalden-Mendes et al., 2011). 

Many of the patients in this study had no access to speech therapy or other types of support therapy. One can explain the significant difference between syndromic and non-syndromic patients regarding access to physiotherapy, occupational therapy, equine therapy and hydrotherapy by the syndromic patients’ additional needs.

Individuals with OFCs are prone to a range of comorbidities, including recurrent otitis, pneumonia, speech delay and otologic and auditory disorders, in addition to emotional and social challenges primarily attributed to facial anatomical alterations. Speech therapy is essential, as it reduces impairment in oral communication and speech delay, which are common conditions among these patients (Gil-da-Silva-Lopes and Monlleó, 2014). In some cases, depending on the severity of the patient’s condition, in particular among syndromic patients, individuals can undergo additional forms of therapy, such as hydrotherapy, occupational therapy and psychotherapy, all aimed at further stimulating and ensuring adequate neuropsychomotor development (Silva *et al.*, 2018).

Typical oral clefts require effort at all healthcare levels due to the high prevalence, and their association with various congenital defects, comorbidities, and the need for long-term treatments. Specialized care is essential for these individuals (Amstalden-Mendes et al., 2007). However, Brazilian studies indicate that health professionals’ knowledge on OFCs is relatively low; many evaluate themselves as not capable to perform routine follow-ups of an individual with OFCs even in their own area of specialization (Gil-da-Silva-Lopes and Monlleó, 2014). 

Based on the data from this study, it is plausible to recommend expanding and enhancing the training and education of healthcare professionals through specific and targeted courses on the subject. Furthermore, establishing more reference centers for care and treatment could facilitate patient access and ensure timely intervention by specialists following diagnosis. Consistent healthcare provided by a specialized team for these children can reduce morbidity and improve quality of life.

State governments, in collaboration with local municipalities, could establish financial assistance programs to subsidize travel and accommodation expenses, or they could improve existing free transportation services for patients and their accompanying family members who need to travel to receive appropriate treatment.

The reference centers should also provide emotional and psychological support to the affected individuals and their families through support groups, meetings, and regular counseling sessions.

Finally, public policies need to be formulated for guidance, education, and raising awareness about orofacial clefts. Public campaigns should be conducted to elucidate treatment procedures, the impacts on patients’ lives, and the significance of access to surgery. The dissemination should be clear, concise, and accessible, possibly through government websites, social media platforms, and even advertising on free-to-air television networks to reach a broader and diverse audience across various age groups.

## Conclusions

The distribution of OFCs by cleft type and gender are greatly similar to those previously described. Low family income, gender, native ancestry and familial recurrence represent risk factors in the investigated cases. There is delay in diagnosis and treatment of OFCs. Using a database, such as the BDCA/CranFlow®/HB/SJRP, can be a good strategy to identify important characteristics that can guide scientific investigations and care policies. 
